# A Case Report of a 61-Year-Old Woman With Jaundice and Cholelithiasis Presenting With Autoimmune Hepatitis

**DOI:** 10.7759/cureus.80829

**Published:** 2025-03-19

**Authors:** Nicholas P Michalakis, Archit Patel, Muhammad Awais

**Affiliations:** 1 Internal Medicine, Piedmont Macon Medical Center, Macon, USA; 2 Graduate Medical Education, Piedmont Macon Medical Center, Macon, USA

**Keywords:** anti-smooth muscle antibody, autoimmune hepatitis, cholelithiasis, ggt, hypothyroidism, jaundice, livedo reticularis, mrcp, r-factor, spider angiomas

## Abstract

Autoimmune hepatitis (AIH) is a complex disease with a chronic cell-mediated immunologic process against healthy liver cells. The clinical presentation can vary since the exact cause of AIH is multifactorial. Here, we report a case of a 61-year-old woman with a past medical history of post-traumatic stress disorder and hypothyroidism who presented clinically with diffuse abdominal distention, nausea, vomiting, jaundice, rash on the torso and legs, and tea-burnt orange urine. The patient underwent an initial workup with a complete blood count (CBC), comprehensive metabolic panel (CMP), and gamma-glutamyl transpeptidase (GGT), with results leading towards a mixed intra- and extrahepatic process. This report will show various findings related to AIH to improve the detection and treatment of these patients early on.

## Introduction

Autoimmune hepatitis (AIH) is a relatively rare disease process with genetic susceptibility or predisposition from various triggers such as environmental or viral factors. The autoimmune mechanism of AIH is supported by cytotoxic T cells and plasma cells seen on histopathology, elevation of specific autoantibodies, and responsiveness with immunosuppressive therapies [1]. AIH causes cell-mediated hepatic injury leading to inflammation and elevation of liver function tests and serum antibodies. AIH is divided into two types based on certain antibodies. AIH type 1 is the most common form of the disease, which predominantly affects women and is prevalent in North America and Northern Europe [1]. The presence of antinuclear antibodies, anti-smooth muscle antibodies, hyperglobulinemia, and certain human leukocyte antigen (HLA) types such as DR3 and DR4 are common in AIH type 1, comprising 90% of all cases. AIH type 2 is less common and is often seen in children and Mediterranean populations. The hallmark antibodies associated with type 2 are anti-liver kidney microsomal antibody-1 (anti-LKM-1) and anti-liver cytosol 1 (anti-LC-1). The exact pathogenesis between the two types of AIH is not completely understood [1]. If AIH goes untreated, life expectancy is 50% at five years, although with treatment, the 10-year survival rate ranges from 80% to 98% [1]. The mainstay management of AIH is initial monotherapy with a corticosteroid (prednisone or prednisolone) or a combination of corticosteroids and azathioprine until remission is achieved [1]. For patients with AIH, symptoms can vary from asymptomatic to nausea, abdominal discomfort, fatigue, and jaundice. Since the symptoms can overlap with many other liver pathologies such as acute hepatitis, chronic hepatitis, primary biliary cirrhosis (PBC), primary sclerosing cholangitis (PSC), and Wilson's disease, it is important to achieve a comprehensive patient history and perform liver function tests and autoantibody testing to establish the diagnosis and guide patient management. The goal of this case report is to add to the literature an unusual presentation of AIH to aid colleagues.

## Case presentation

A 61-year-old Caucasian woman with a past medical history of hypothyroidism and post-traumatic stress disorder (PTSD) presented to our hospital with complaints of diffuse abdominal pain and distension of at least nine weeks duration as well as nausea and vomiting for one week. The patient also complained of yellow discoloration of her skin over most of her body, worse in the upper half of her torso, as well as her eyes for the past two days prior to presentation. In addition to the above, the patient also endorsed tea-burnt orange-appearing urine and increased urinary frequency. The patient stated that her pain was diffuse in distribution from just beneath her sternum to slightly above her bladder and was worse in intensity in the center of her abdomen. It was exacerbated in the postprandial state as well as with deep inspiration. The pain was characterized as dull and aching in nature and occasionally woke her up from her sleep. It was attenuated with emptying of the bladder. Nausea and vomiting began a week prior, was sudden in onset, and had been intractable with the patient unable to keep down solids, liquids, or medication. The jaundice and scleral icterus were first noticed by the patient's daughter. The patient had not experienced this constellation of symptoms in the past. She denied any recent travel.

The patient sought medical evaluation the week prior to her hospitalization at an urgent care facility for her abdominal pain and dark-colored urine. Tests performed at the urgent care included a urinalysis and urine culture. Urinalysis showed clear, dark-yellow urine with large levels of bilirubin. The urine culture showed mixed flora consistent with contamination. The patient also had an X-ray of her abdomen which was positive for moderate fecal retention and cholelithiasis in the right upper quadrant. The patient's symptoms had compounded since the visit to the urgent care prompting the patient to undergo further evaluation at our hospital. 

Upon further questioning, it was revealed that the patient has a family history of pancreatic cancer in one of her siblings. Furthermore, the patient stated that she has a personal history of intravenous (IV) drug and alcohol use. The patient denies recent IV drug or alcohol use and states that she has not consumed alcohol since 2012. No signs of alcohol withdrawal were seen. An ethanol level and urine drug screened were not ordered as part of the workup. The patient also denies taking any new medications or over-the-counter (OTC) supplements.

The review of systems for our patient was unremarkable except for shortness of breath with deep inspiration, abdominal pain, constipation, nausea with vomiting, increased urinary frequency, and tea-burnt orange-colored urine. In addition, the patient endorsed skin changes such as discoloration in her lower extremities, jaundice of her skin, and yellowing of her eyes.

**Figure 1:**
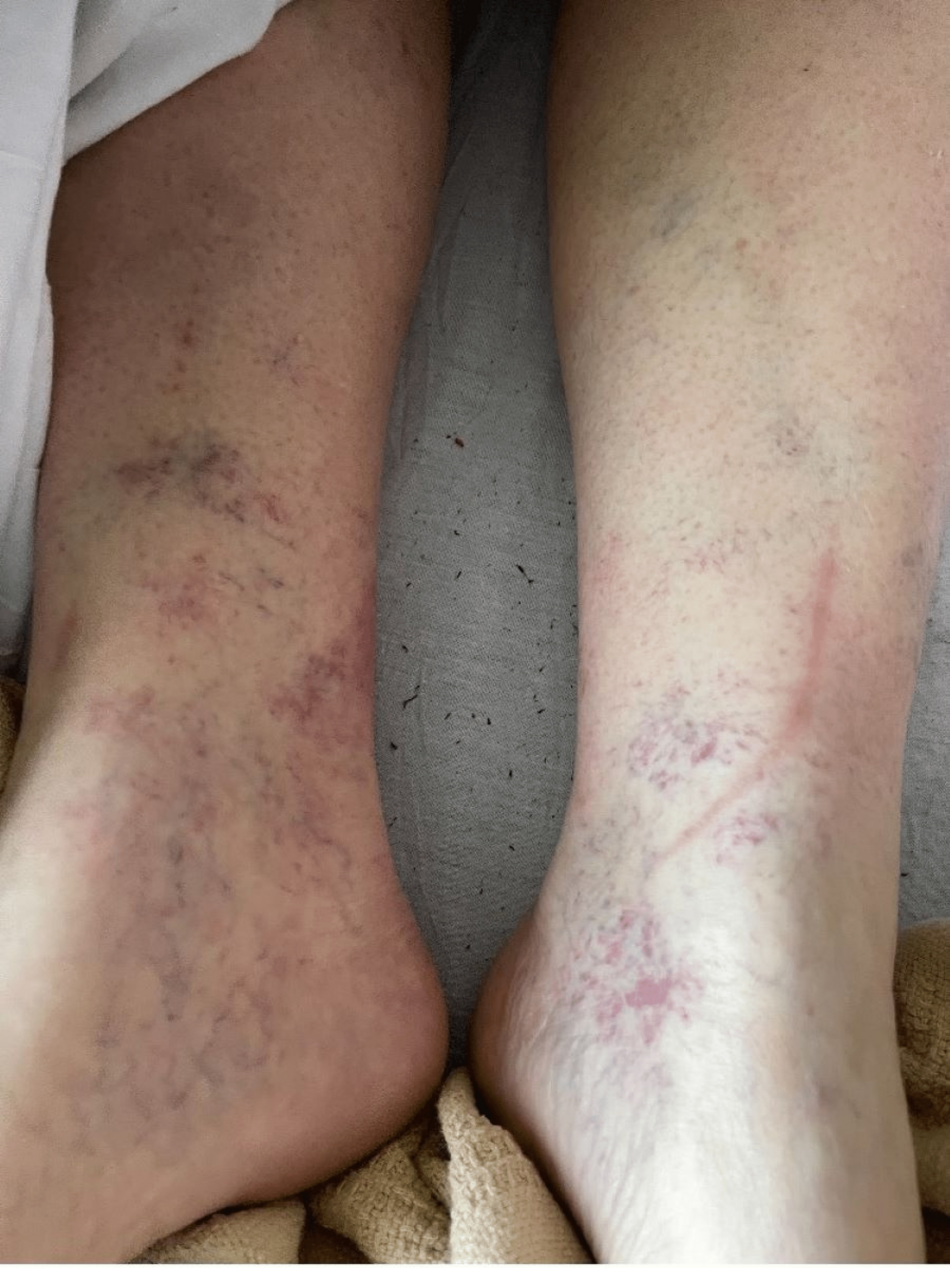
Scattered clusters of erythematous vascular macules on the bilateral ankles and feet

**Figure 2:**
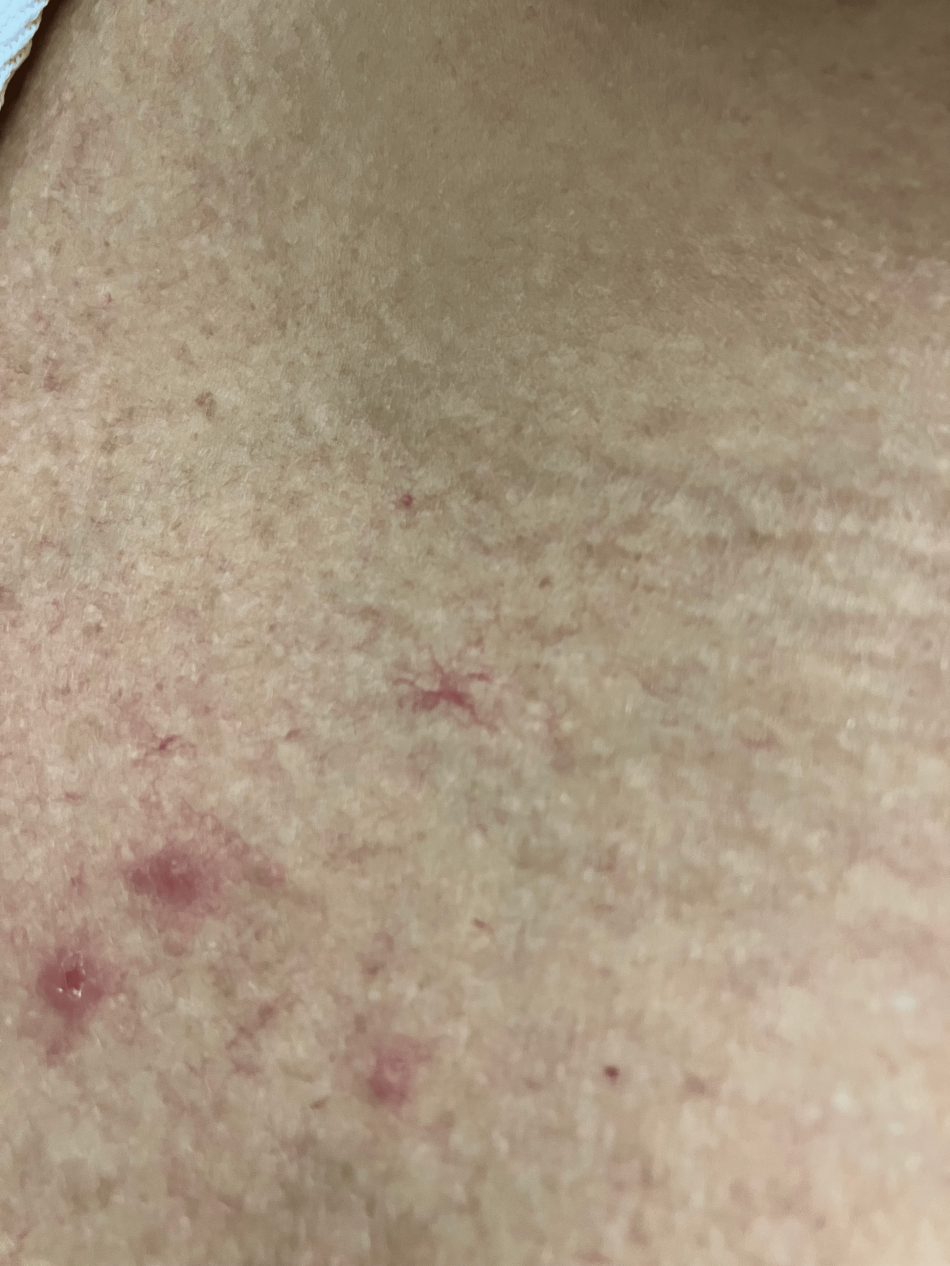
Small multiple spider angiomas on the chest

**Figure 3:**
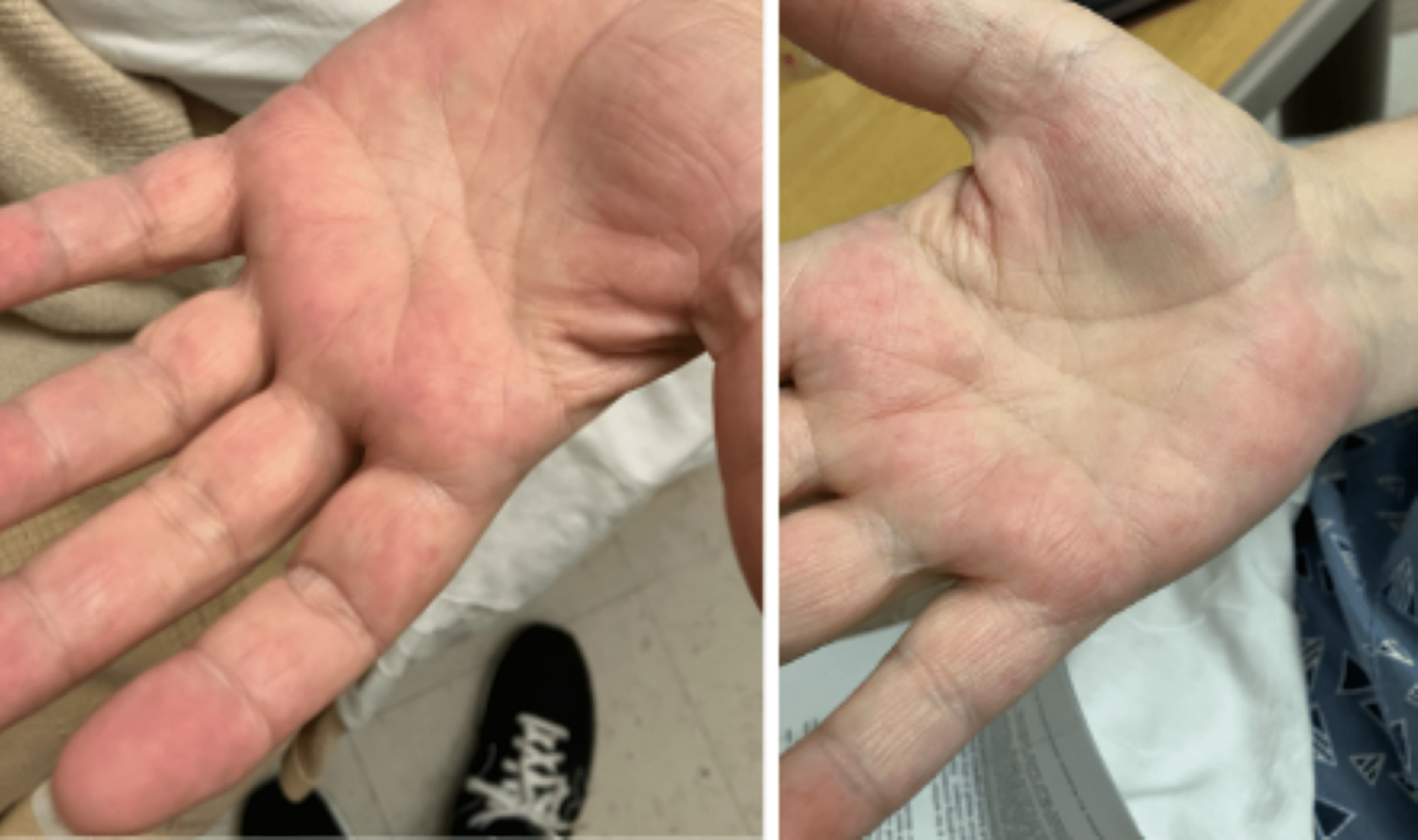
Multiple faint macules and patches of erythema on the bilateral hands

On physical examination, a generalized, mild jaundice was noticeable, more so on the patient's upper extremities and abdomen. On the patient's bilateral lower extremities, there appeared to be a lacy rash similar in appearance to livedo reticularis (Figure 1). There were obvious varicose veins and signs of venous insufficiency surrounding the patient's ankles, bilaterally. On the upper torso, most noticeably on the patient's chest, were multiple minute 0.5 cm or smaller spider angiomas (Figure 2). On the inside of the patient's palms, there were multiple faint macules and patches of erythema present (Figure 3). Lastly, on examination of the patient's eyes, bilateral scleral icterus was appreciated.

Following the patient's admission for painless jaundice to our hospital, a thorough workup was initiated. Initial workup for the patient included a comprehensive metabolic panel (CMP) and complete blood count (CBC), with differential. The CMP was remarkable for a mildly elevated alkaline phosphatase (ALP) of 141 U/L (normal values between 46 and 116 U/L), markedly elevated total bilirubin of 6.9 mg/dL (normal value 0.2-1.3 mg/dL), as well as elevated aspartate aminotransferase (AST) of 732 U/L (normal value <34 U/L) and alanine transaminase (ALT) of 755 U/L (normal values between 10 and 49 U/L) [2]. A direct bilirubin was ordered and found to be raised at 5.00 mg/dL. A gamma-glutamyl transferase (GGT) test was ordered which came back elevated at 191 U/L (normal range is 0-30 U/L) [3].

In addition to serum lab tests, inpatient imaging of our patient was performed to rule out any possible hepatobiliary and pancreatic obstruction or inflammation. An X-ray of the abdomen was done as an outpatient prior to the patient's hospitalization which revealed moderate fecal retention and possible gallstones. Inpatient imaging included an ultrasound of the abdomen (US of the abdomen), followed by magnetic resonance cholangiopancreatography (MRCP). MRCP was also chosen to evaluate for pancreatic cancer given the patient's family history.

**Figure 4:**
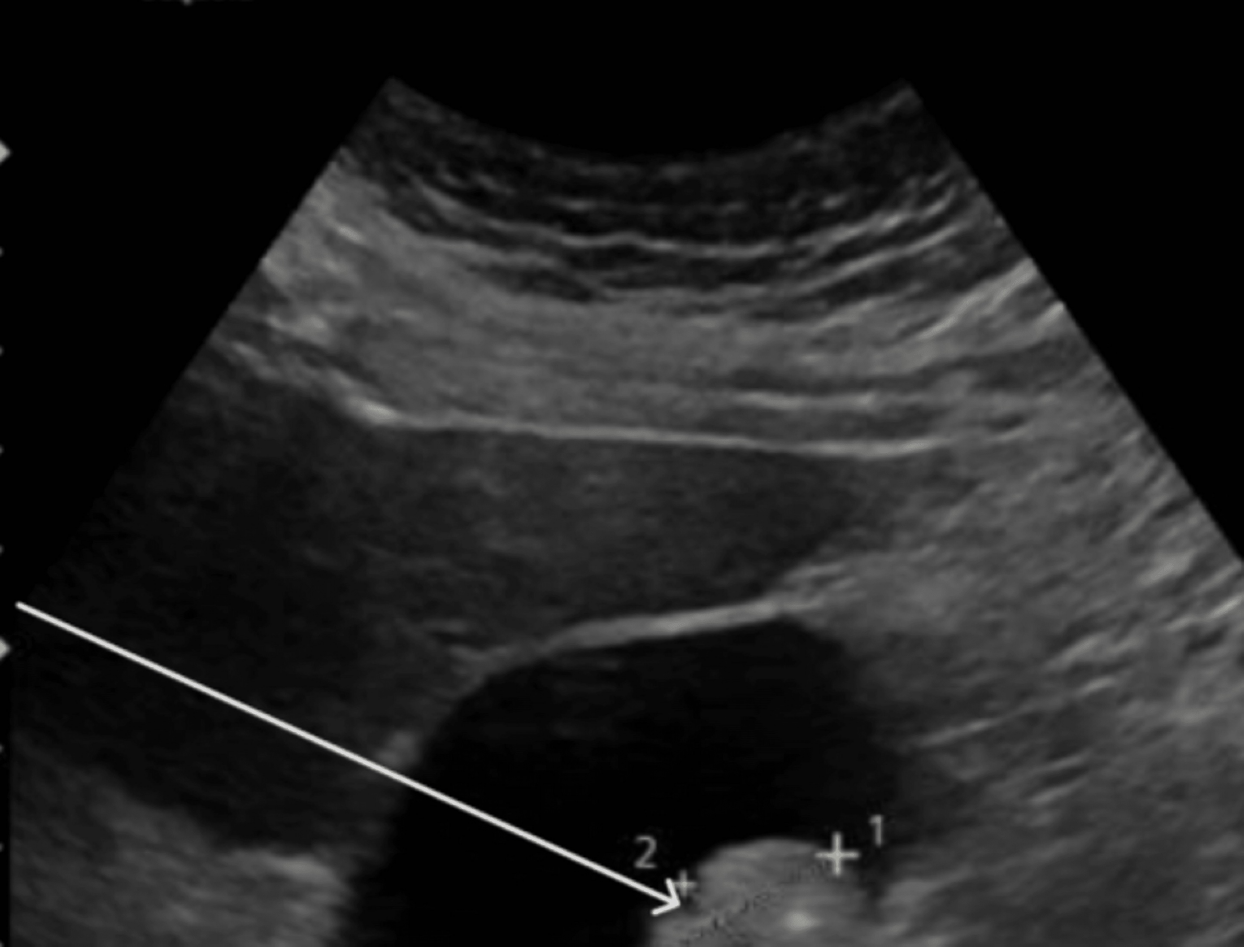
Cholelithiasis (white arrow) with no evidence of acute cholecystitis

The US of the abdomen showed evidence of hepatomegaly, although no intrahepatic biliary dilation was found. In addition, the main portal vein appeared patent and normal. Hepatopetal flow was appreciated. The gallbladder appeared mostly unremarkable with no gallbladder wall thickening or pericholecystic fluid. The common bile duct was visualized and was of normal caliber with a negative sonographic Murphy's sign. There was however some biliary sludge as well as gallstones in the gallbladder, although no evidence of acute cholecystitis was appreciated (Figure 4).

**Figure 5:**
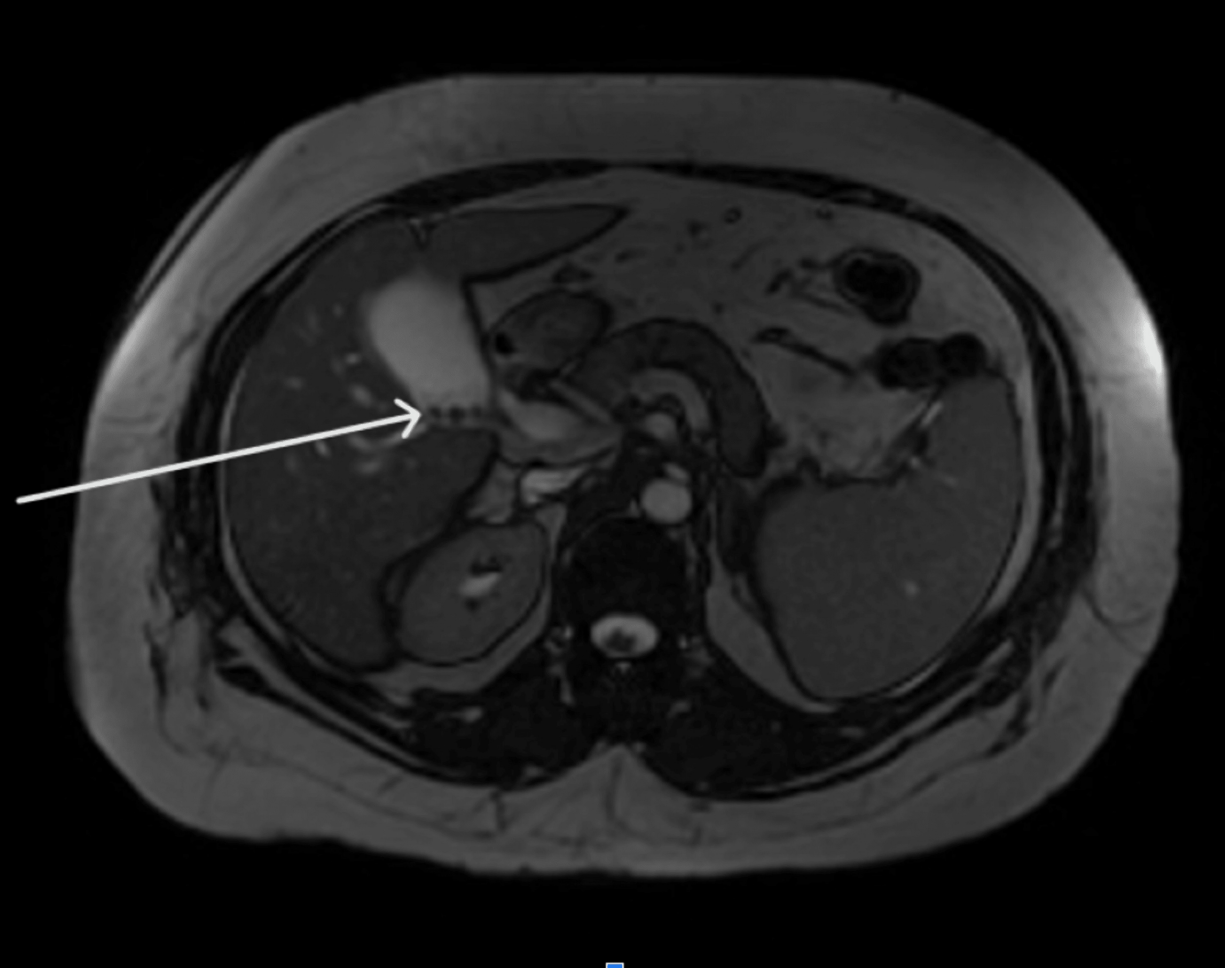
Cholelithiasis (white arrow) with no choledocholithiasis or ductal dilatation. The pancreas appears uniform in structure, without apparent focal lesions or ductal dilations

The MRCP with and without contrast was done after the US of the abdomen. Results stemming from the MRCP were largely unremarkable. The liver was visualized as normal in morphology without evidence of hepatic or iron deposition. There was no evidence of suspicious focal masses nor intrahepatic or extrahepatic biliary ductal dilation. In addition, there were no obstructing stones or lesions present. The gallbladder was visualized and was only positive for cholelithiasis without discrete wall thickening. The pancreas was visualized and was grossly normal in appearance (Figure 5).

Following a plethora of additional labs, including an autoimmune panel, the patient was tentatively diagnosed with autoimmune-induced liver injury, presumed to be AIH, and started on prednisone. The patient endorsed improvements in her symptoms in the ensuing days following the start of her treatment. She was subsequently discharged home on a prednisone taper and strongly counseled on following up with a gastroenterologist as an outpatient for definitive evaluation and continued management.

## Discussion

AIH is a fairly uncommon, non-resolving, chronic inflammatory liver disease characterized by hypergammaglobulinemia (elevated IgG), autoantibodies such as anti-smooth muscle antibody (anti-SMA), anti-nuclear antibody (ANA), and anti-liver kidney microsome (anti-LKM), as well as interface hepatitis on histology [1]. AIH is particularly challenging to diagnose owing to its shared signs, symptoms, and overall clinical presentation which appear similar to and overlap with other liver diseases, specifically cholestatic liver diseases such as drug-induced liver injury (DILI), PSC, PBC, non-alcoholic steatohepatitis (NASH), alcoholic steatohepatitis (ASH), and viral hepatitis. AIH is a clinical diagnosis that relies on multiple lab tests as well as liver biopsy findings for a definitive diagnosis. Oftentimes, even with numerous pertinent, positive tests, the diagnosis may still be inconclusive. In spite of the diagnostic challenges, AIH has a favorable prognosis with treatment with immunosuppressive therapy being the mainstay. Untreated AIH has a poor prognosis with liver failure and cirrhosis being the main complications. The disease however is non-curable, with recurrent relapses even after remission has been achieved [1].

AIH is a relatively rare liver disorder with a prevalence ranging from 16-18 cases per 100,000 to as high as 42.9 cases per 100,000, depending on the country and ethnic background [1]. The disease affects both sexes in a 3:1 female-to-male ratio. AIH can strike at any age, although a bimodal distribution is often seen with peak incidence occurring during childhood and adolescence, as well as between the fourth and sixth decades of life. Clinical appearance of AIH varies with some patients experiencing mild, non-specific symptoms such as fever and a maculopapular rash, while others experience signs and symptoms found normally with chronic liver disease such as spider angiomas, palmar erythema, and jaundice (as seen in our patient). AIH type 1 accounts for roughly 90% of cases, with AIH type 2 accounting for the rest. AIH type 1 is usually characterized by elevated ANA and/or anti-SMA, while AIH type 2 features anti-LKM-1 or anti-LKM-3. AIH can sometimes overlap with certain cholestatic diseases such as PBC and PSC as variant forms although these are less common than either of the diseases on their own [1].

Diagnosing AIH

Diagnosis of AIH is typically clinical and involves the exclusion of other liver diseases. The differential diagnosis of AIH is fairly large, and there are many other conditions that need to be considered during the workup. These disease states include PBC, PSC, ASH, NASH, DILI, such as with acetaminophen toxicity, as well as viral hepatitis such as with hepatitis B and C as well as Epstein-Barr virus (EBV) [1]. A workup for jaundice typically ensues such as with our patient. Workup often begins with a CMP, to evaluate liver transaminases. Our patient's CMP was remarkable for an ALT of 755 U/L (normal values between 10 and 49 U/L) and an AST of 732 U/L (normal value <34 U/L). ALP was only slightly elevated at 141 U/L (normal value between 46 and 116 U/L) [2]. Total bilirubin was markedly elevated at 6.9 mg/dL (normal value 0.2-1.3 mg/dL). The larger AST and ALT values compared to the ALP revealed that our patient's signs and symptoms were more hepatocellular than cholestatic in nature [3]. An additional test, the R-value also known as the R-factor, aided in differentiating whether the elevated aminotransferases were due to hepatocellular or cholestatic injury. The R-value is equal to (ALT/upper limit of the normal ALT value)/(ALP/upper limit of the normal ALP value). A score of 5 or greater suggests elevated aminotransferases are due to hepatocellular injury, a score between 2 and 5 indicates a mixed pattern of injury (hepatocellular and cholestatic), and a score of 2 or less is indicative of a cholestatic pattern of injury [3]. Our patient's R-value was calculated to be 16.5 which was suggestive of elevated aminotransferases secondary to hepatocellular insult.

Once we confirmed that the patient's aminotransferases and total bilirubin were elevated (6.9 mg/dL), we needed to determine if the patient's jaundice was caused by unconjugated (indirect), or conjugated (direct), hyperbilirubinemia. Unconjugated or indirect hyperbilirubinemia is caused by increased bilirubin production such as with red blood cell destruction, as seen in glucose-6-phosphate dehydrogenase deficiency (G6PD-deficiency), or due to impaired bilirubin uptake in the liver and/or hindered bilirubin conjugation as seen in Gilbert syndrome. Conjugated or direct hyperbilirubinemia can arise from disorders such as PBC, viral or alcoholic hepatitis, as well as inherited disorders such as Rotor syndrome and Dubin-Johnson syndrome, leading to intrahepatic cholestasis and hepatocellular damage or through extrahepatic cholestasis such as with PSC and choledocholithiasis [1]. Recall that the direct bilirubin came back elevated at 5.00 mg/dL which indicated that the patient had conjugated hyperbilirubinemia [4,5]. For the sake of completeness, a GGT test was ordered which came back elevated at 191 U/L (normal range is 0-30 U/L) [6]. The elevated GGT, the R-value >5, and the markedly elevated AST and ALT compared to the ALP helped narrow our differential more towards intrahepatic cholestasis. 

Our patient did endorse past alcohol use, although she stated that she had not had any alcohol since 2012 and has not had an episode of jaundice or scleral icterus in the past which lessened the likelihood of this being due to alcoholic hepatitis. Neither an ethanol level nor a urine drug screen was ordered and checked, although this could have been done on admission to ensure a more thorough workup considering the patient's past substance use, as well as the fact that both illicit substances (such as methamphetamines and cocaine) and non-illicit substances such as alcohol can lead to hepatic injury. However, as far as alcohol-induced hepatitis is concerned, our patient's AST/ALT ratio was less than 2 (0.97). In alcoholic hepatitis, one would expect to see an AST/ALT ratio of 2 or greater. In addition, our patient's severely elevated AST and ALT levels further decreased the probability of alcoholic hepatitis from being our primary diagnosis, as one would expect aminotransferase levels of 300 U/L or less [7-14]. Other potential causes of hepatitis, including DILI, such as with acetaminophen toxicity, and viral hepatitis such as with hepatitis A, B, and C and EBV, were explored although testing yielded negative results [13-16]. NASH and infiltrative diseases such as Wilson's disease, sarcoidosis, and hemochromatosis were also investigated. Given the patient's normal ceruloplasmin, lipid, and iron levels, these were understandably not high on the differential [1].

In addition to labs, imaging may also be employed for diagnostic workup on jaundice and suspected liver injury. MRCP is especially useful in evaluating causes of intra- and extrahepatic cholestasis such as PSC and choledocholithiasis, respectively. Choledocholithiasis describes the presence of biliary stones in the common bile duct. It is mainly diagnosed with imaging via MRCP following elevated aminotransferases and GGT [17,18]. PSC is characterized by fibrosis, inflammation, and narrowing of intrahepatic and extrahepatic bile ducts. It is also denoted by elevated serum aminotransferases, especially that of ALP greater than four times the upper limit of normal which was not characteristic of our patient [19,20]. MRCP is also particularly helpful in evaluating liver injury secondary to infiltrative and iron deposition diseases such as hemochromatosis, Wilson's disease, and alpha-1 antitrypsin deficiency, all of which were not evident in our patient [1].

Following the negative viral hepatitis and largely unremarkable abdominal imaging results, the next step was to determine if there was an autoimmune component at play such as AIH or PBC. Initial autoimmune labs included an ANA immunofluorescence assay (IFA) with a reflex titer, and anti-SMA and anti-mitochondrial antibody tests were ordered to evaluate for AIH and PBC, respectively [21-23]. The ANA IFA screen came back positive along with an ANA titer of 1:40 which is considered a positive result in adults [1]. The positive ANA IFA and titer helped confirm that our patient's condition was indeed autoimmune in nature although it is not specific to any one condition [24]. The anti-SMA came back elevated while anti-mitochondrial antibodies came back negative for our patient, making the diagnosis of PBC less likely. The anti-SMA test being positive increased the likelihood of the hepatocellular injury being due to AIH [23]. Another confirmatory test for AIH is immunoglobulin G (IgG) which we measured in our patient. IgG came back markedly elevated at >2500 mg/dL (normal levels are between 600 and 1540 mg/dL). Elevated IgG can also be found in patients with chronic liver disease, although the addition of a positive anti-SMA helped distinguish AIH from non-autoimmune-related chronic liver disease states [25]. At this point in our patient's workup, AIH was the most likely etiology of her intrahepatic cholestasis and hepatitis. A liver biopsy could have also been performed although it is not necessary given the elevated IgG levels and the fact that the anti-SMA came back positive. Liver biopsy is usually reserved in instances where AIH is suspected and the results of the autoantibodies and/or IgG levels are equivocal [26]. With a diagnosis of AIH confirmed, the last step was to differentiate between AIH type 1 and type 2. To do this, we ordered an anti-LKM-1 test which is present in roughly 70% of patients with AIH type 2 and, as an aside, in 10% of patients with hepatitis C [27-29]. Our patient's anti-LKM-1 came back negative, decreasing the probability of AIH type 2 as the diagnosis. Anti-liver cytosol antigen-1 (anti-LC1), another autoantibody found to be positive in approximately 30% of patients with AIH type 2, was not checked. While anti-SMA and anti-LKM-1 are the more common autoantibodies used to diagnose AIH, there are other antibodies such as anti-soluble liver antigen/liver-pancreas (anti-SLA/LP) antibody, which is the most specific marker for the disease, although its presence is found in both AIH types 1 and 2 [1].

There are diagnostic criteria and a scoring system proposed by the International Autoimmune Hepatitis Group (IAIHG), since 1993. The IAIHG scoring system looks at whether specific criteria are present such as ANA and AIH-specific autoantibodies (such as SMA, LKM, and SLA/LP), as well as their titer level (the higher the titer, the greater the score), and the level of IgG. The scoring system also considers liver biopsy results, whether or not there is histology that is compatible with AIH present (such as interface hepatitis, cytotoxic T cells, plasma cells, etc.), and, lastly, the absence or presence of viral hepatitis. A score of 7 or higher implies definite AIH, while a score of 6 or higher suggests probable AIH [1]. Our patient certainly met many of the diagnostic criteria such as elevation of at least one type of serum aminotransferase, elevated IgG, and/or ANA and SMA at a titer of at least 1:40. In addition, we were able to exclude other liver diseases with a similar presentation to AIH such as DILI, alcoholic liver disease, and viral hepatitis. Our patient would have achieved a score of approximately 5. This is due to a lack of a liver biopsy to examine the liver histology, as well as a lack of examination for SLA/LP autoantibodies. 

Treatment of AIH

Treatment of AIH is often lifelong and centers on achieving complete remission while preventing further advancement of the disease and irreversible hepatic insults. Glucocorticoids, such as prednisone and prednisolone, and anti-metabolites, such as azathioprine, are the primary treatment modalities to attain remission in AIH. This requires indefinite maintenance therapy. A small subset of patients can remain in remission even after treatment withdrawal. The prognosis for untreated moderate to severe AIH is poor with disease progression leading to cirrhosis, liver failure, and even hepatocellular carcinoma [1].

Some patients with mild or asymptomatic AIH may undergo spontaneous resolution of their disease state. Most, however, eventually become symptomatic and experience disease progression. Current AIH guidelines recommend immunosuppressive induction therapy for all patients with active disease. There have been a multitude of studies that highlight the survival benefits of immunosuppressive induction therapy by comparing mortality rates. These studies compared mortality rates between corticosteroids (prednisone/prednisolone) and combined corticosteroid plus azathioprine, azathioprine alone, and placebo. Corticosteroids alone or in combination with azathioprine showed similar mortality rates of 6% and 7%, respectively, while the azathioprine group alone had a mortality rate of 36%. The placebo group, as one would expect, had the highest mortality rate of 41%. Combination therapy was associated with fewer side effects with values of 10% versus 44% for the corticosteroid-azathioprine group versus the corticosteroid group alone, respectively [1].

Therapeutic strategy for remission induction starts with corticosteroids, such as prednisone or prednisolone at 0.5-1 mg/kg/day (typically 40-60 mg/day). In patients with a positive response, azathioprine is added as a steroid-sparing therapy and gradually increased up to 1-2 mg/kg/day for maintenance dosing. Typical starting doses of azathioprine are around 50 mg. This medication addition generally occurs between weeks 2 and 3 which is typically when serum aminotransferases and IgG are checked for response to glucocorticoids. Bilirubin levels ideally should be below 6 mg/dL (as azathioprine can increase bilirubin levels and rarely cause hepatotoxicity). Azathioprine therapy can be started earlier in an attempt to wean the patient off corticosteroids in cases where steroid side effects are of immediate concern, such as in post-menopausal women, osteoporosis, diabetes, etc. As azathioprine is started and titrated up over time, corticosteroids should be tapered down 5-10 mg every 1-2 weeks until biochemical remission is achieved and maintained on azathioprine which carries fewer long-term side effects than corticosteroids. A liver biopsy or FibroScan to evaluate for histological remission is not generally required unless there are specific concerns such as poor response to immunosuppressive treatment, treatment side effects, or other extenuating circumstances [1].

While the goal of immunosuppressive therapy is total clinical, biochemical, and histological remission with treatment withdrawal, most patients experience relapse and will need to be restarted on maintenance treatment. Approximately 80-90% of patients show improvement in their serum aminotransferase levels after the initiation of immunosuppressive treatment. A minority of patients (20%) may achieve long-term remission even after maintenance treatment is withdrawn. A smaller percentage of patients with AIH experience little to no response to immunosuppressive therapy which may be indicative of lack of treatment adherence and even possible misdiagnosis. A small subset of patients with fulminant or acute-severe AIH may also exhibit poor treatment response. These patients would benefit from prompt referral to a transplant center for possible liver transplant given their acute liver failure [1].

Most patients with AIH respond well to immunosuppressive therapy and enter remission. Remission can be measured in the biochemical sense (normalization of serum aminotransferases and IgG), via liver biopsy (no longer see interface hepatitis on histology), or both. As one would expect, checking for remission biochemically is the easiest and preferred method. Lack of normalization of serum aminotransferases or persistent elevations could indicate relapse after treatment is withdrawn and/or disease progression to cirrhosis, all of which portend worse outcomes [1].

One of the challenges in treating AIH is a lack of consensus on optimal treatment duration. Typically, treatment should be ongoing for a minimum of three years. Furthermore, treatment should continue for at least two years from the point of biochemical remission (normalization of IgG and serum aminotransferase levels) [1]. Patients should be monitored closely with serial serum aminotransferase levels checked as immunosuppressive medications are weaned down and eventually totally withdrawn. Relapse is a common phenomenon in AIH, with about 50-90% of patients relapsing within the first year of treatment withdrawal. In other patients, relapse can occur many years later, underscoring the importance of continual liver lab monitoring. Relapse is usually heralded by a return of clinical symptoms of cholestasis (jaundice, scleral icterus, palmar erythema, etc.), as well as serum transaminase levels (ALT) greater than three times the upper limit of normal. A liver biopsy is typically not needed. Treatment consists of the same regimen that led to remission. Earlier detected relapses can be treated with lower doses of immunosuppressive therapy to induce remission. Patients with multiple relapses are recommended to remain on maintenance treatment indefinitely [1]. Continuous maintenance therapy with azathioprine is the preferred treatment in patients in remission. The dose of azathioprine can be gradually titrated up to 2 mg/kg/day. Azathioprine is able to maintain remission for as long as the patient is taking the medication (83% remission rate over a 67-month period) [1]. It is the preferred choice if one is concerned about steroid-induced side effects such as hypertension, osteoporosis, worsening diabetes, etc. Patients on azathioprine need to be monitored for cytopenia and increased risk of malignancy. For patients intolerant of azathioprine, corticosteroids such as prednisolone can be used at the lowest possible dose that is able to maintain the patient in remission. Frequent monitoring of serum aminotransferase levels and IgG is paramount. During remission induction, while the patient is on corticosteroid and azathioprine, the patient's clinical and lab markers should be monitored every four weeks. As steroids are tapered down, this monitoring can be extended to every 1-3 months. Finally, while on maintenance treatment, monitoring labs every 3-6 months is acceptable [1].

Prognosis

Despite AIH being a chronic, non-curable disease with relatively high relapse rates (50-90%), a large percentage of patients (over 80%) on induction therapy reach remission within two years. Without treatment, life expectancy is poor at roughly 50% at five years and 10% at 10 years. With maintenance treatment, life expectancy is 90% at 10 years and over 70% at 20 years [1].

## Conclusions

This case report highlights the significance of broadening one's differential and considering autoimmune etiologies such as AIH when evaluating for causes of intrahepatic cholestasis and hepatitis. It is especially of importance in older, female patients with pre-existing autoimmune conditions such as in our patient. While AIH could not be diagnosed with absolute certainty in our patient, due to a mild ANA titer and lack of a liver biopsy displaying the tell-tale signs of interface hepatitis on histopathology, many of our patient's other labs signaling AIH, such as IgG and anti-SMA, were either positive or elevated. Our patient's disease remission at the start of her prednisone therapy and subsequent relapse while tapering down her steroid pointed towards an autoimmune disease of the liver highly suggestive of AIH. AIH can be challenging to treat. While remission is possible, patients often relapse, necessitating restarting immunosuppressive therapy. In addition, patients need intermittent clinical and lab value (serum aminotransferases and IgG) monitoring. This case report serves as a reminder to keep AIH on the differential when working up liver disease. Furthermore, it aimed to outline several of the more pertinent guidelines for treatment. Our patient continues to do well. The patient has been in remission, successfully weaned off of prednisone, and has been on azathioprine therapy which she is tolerating.
